# Groundwater quality with special reference to fluoride concentration in the granitic and basaltic contact zone of southern India

**DOI:** 10.1016/j.dib.2020.106462

**Published:** 2020-10-28

**Authors:** Edukondal Allam, Hari Krishna Gangula, Ramalingaiah Arukonda, M. Muralidhar

**Affiliations:** Department of Geology, Osmania University, Hyderabad 500007, India

**Keywords:** Granitic-basaltic contact, Semi-arid region, Fluoride contamination, Groundwater, Telangana

## Abstract

The main focus of this data article is to evaluate the groundwater and surface water quality from a granitic-basaltic watershed in a semi-arid region. The obtained values are evaluated concerning the drinking water quality standards proposed by WHO, specifically for the semi-arid regions. All the physio-chemical parameters (fourteen) required for the calculation of water-quality indices and source appreciation were derived. Person correlation analysis for the measured parameters is presented with high to poor correlation groups in the study region. A brief description of the methods and calculation of water quality indices is mentioned. The data can be re-used to calculate to evaluate the suitability for drinking and agriculture needs of the basin; besides, it can be helpful to the authorities to make policies to mitigate the water quality vulnerability.

**Specifications Table**

**Table:** 

Subject	Earth and Planetary Sciences
Specific subject area	Hydro-Geochemistry
Type of data	Tables, Figures, and Graphs
How data were acquired	The hand GPS is used to mark the sample locations, and in-situ measurements were made using the portable pH, electrical conductivity (EC), Total dissolved solids (TDS) meters. For major ions, i.e., Calcium (Ca_2_^+^), Magnesium (Mg_2_^+^), Sodium (Na^+^), Potassium (K^+^), Bicarbonate (HCO_3_^−^), Carbonate (CO_3_^2−^), Chloride (Cl^−^), Fluoride (F^−^), Sulphate (SO_4_^2−^) and Nitrate (NO_3_^−^) were measured using the Ion chromatography. All the Water Quality Indices were calculated using defined formulas. Grapher-13 and ARC GIS 10.3 tools were used for producing maps and graphs.
Data format	Raw (in-situ measurements), Filtered and Analyzed (lab measurements)
Parameters for data collection	Both groundwater (tube wells, bore-wells) and surface water samples were collected in two-liter bottles and stored in refrigerators under specified conditions until the analysis.
Description of data collection	Fifty samples i.e., 42 groundwater, and 8 surface water samples were collected in pre (May-2015) & post (December-2015) monsoon periods, respectively.
Data source location	Jukkal and Bichukunda watershed is located in the western part of Nizamabad District, Telangana, India.
Data accessibility	Available with the article. The total data of fourteen water quality parameters in the post and pre-monsoon seasons are provided in the supplementary document.

**Value of the Data**

• The present data is first reporting from the granitic-basaltic contact zone of a semi-arid region using the various water quality indices and possible controlling mechanisms.
• The data deals with the water quality, which reveals the hydro-geochemical nature of the available water resources and how far these are suitable for drinking and irrigation purposes.
• The present data provide baseline information in fewer studies semi-arid region; thus it can be used for researchers for making a water-rock interaction model and also useful to government and non-governmental organizations to adopt effective planning methods and mitigation.

## 1 Data Description

### 1.1 Study area

**Fig. 1:**
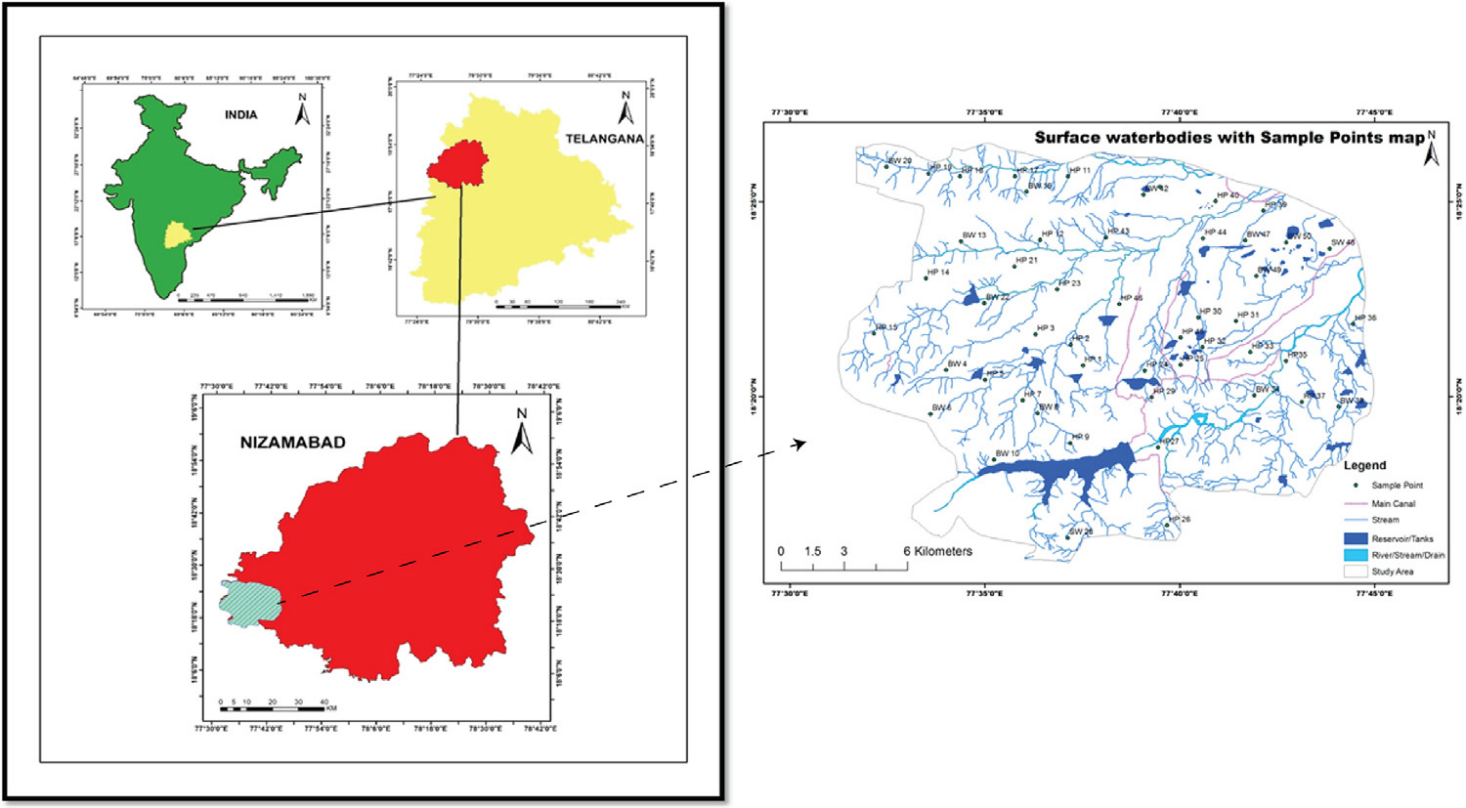
Location map of the study area showing sample locations and drainage pattern.

Jukkal and Bichukunda watershed is situated in the western part of Nizamabad District and falls in the Survey of India topo sheet no. E 43L11. This region lies between Longitude 77° 30ˈ−77° 45ˈ and latitudes 18° 30ˈ −18° 15ˈ with an aerial extent of 355 km^2^ (Fig. 1). The watershed is situated in the Manjira river basin, a tributary of the Godavari river. The drainage pattern of the watersheds is dendritic and sub dendritic (Fig. 1), and the region is situated at an altitudinal ranging from 370 to 500 m above mean sea level (AMSL). Normal average annual rainfall estimated in Jukkal and Bichukunda regions during 2015 is 713 and 412 mm, respectively, clearly indicating the semi-arid climate [1]. The area is hot for most of the year, i.e., during summer (May), the maximum temperature is around 41–45 °C, and the minimum temperature is around 20–24 °C in winter months with an average annual temperature of 29.5 °C [2].

Geologically, the study area covers a part of the stable southern Indian shield consisting of peninsular gneissic complex (PGC) and Deccan Traps (Fig. 1; [3]). The region is occupied with a well-developed soil cover represented by reddish-brown color in granite dominated region, lomey and black, or regur soil color, especially in the basaltic region. It is also reported that the soils in the study area are relatively permeable and can absorb most of the rainwater through infiltration except during intensive rains [4]. Groundwater occurs in the soil of weathered granites and basalts and semi weathered fractured zones in semi-confined conditions and, the average depth of groundwater is about 8–10 m in the study region [5]. The region occupied by granite rocks possesses negligible primary porosity; however, the part under landed by Deccan Traps are in the phreatic condition in the weathered zone above the hard rock, and semi-confined condition in the region dominated by the fissures, fractures/joints [2].

### 1.2 Data

**Fig. 2:**
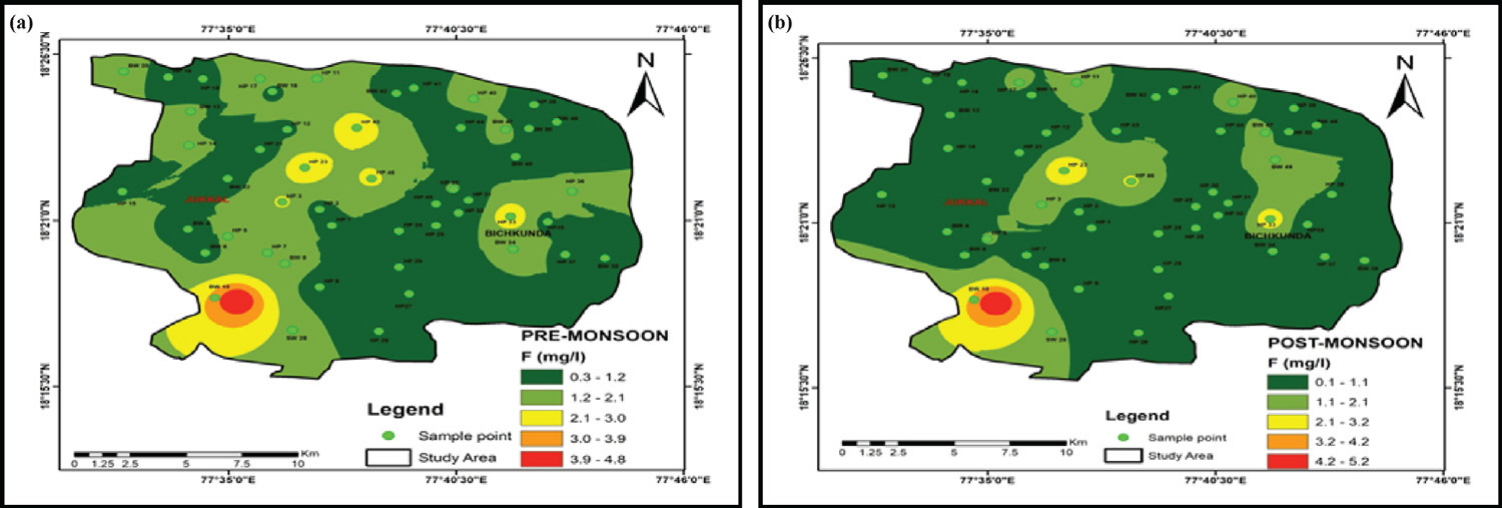
Spatial distribution map of fluoride in (a) pre-monsoon, and (b) post-monsoon seasons.

**Fig. 3:**
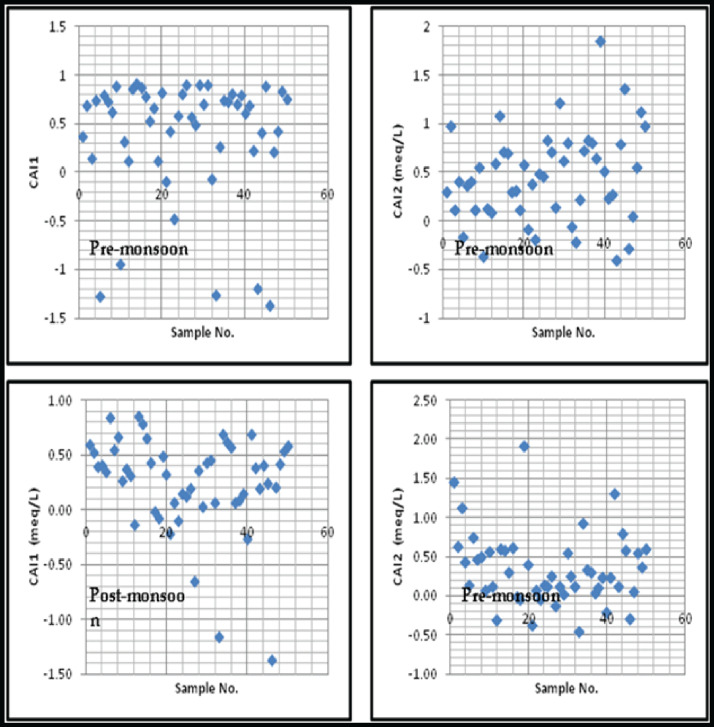
Chloro-Alkaline Indices (CAI) graph for pre and post-monsoon seasons.

**Fig. 4:**
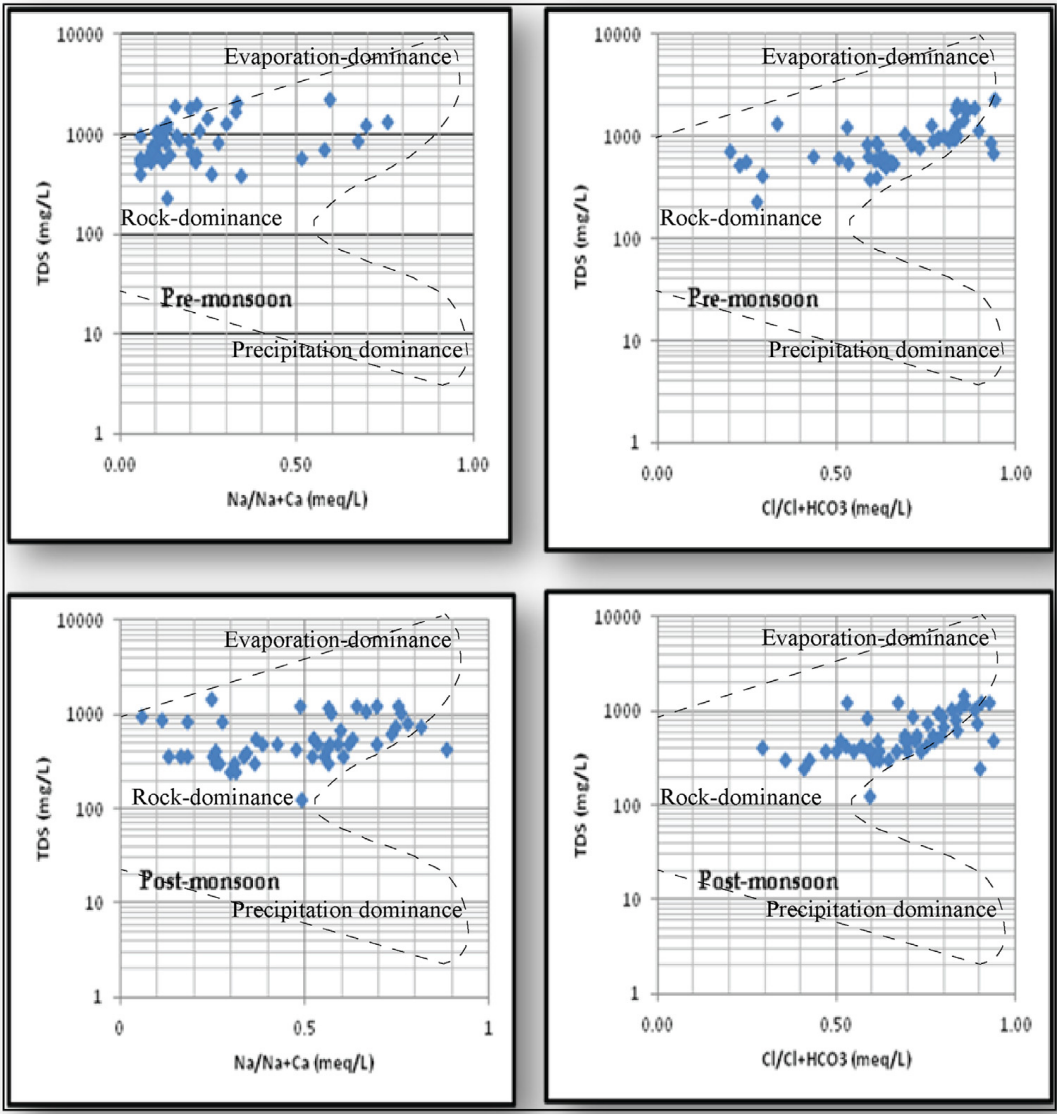
Gibbs plot for the water samples in pre and post-monsoons seasons.

Descriptive statistics of the measured water quality parameters of the collected samples were presented in Table 1 [6,7]. The spatial and temporal distribution of fluoride in the watershed is presented in Fig. 2. The granite-basalt contact zone (southwest) part is showing the high fluoride concentration in both the pre and post-monsoon seasons. The fluoride concentrations are ranging from 0.35–4.84 mg/l with an average of 1.32 mg/l, 0.17–5.22 mg/l with an average of 1.01 mg/l in pre and post-monsoon seasons, respectively. The fluoride concentrations in the post-monsoon season are higher than the monsoon season. Table 2 and Fig. 3 details the calculated Cholro-Alkaline Indices values of the samples. The data was plotted on Gibbs plot (Fig. 4) to establish the relationship of water composition and aquifer lithological characteristics. Water quality classification based on WQI values of the study area was depicted in Table 3 and Fig. 4. Table 3a, 3b provides the details of inter-relations among the measured parameter using the person correlation matrix.

**Table 1:** Statistics of physical and chemical parameters of groundwater samples in pre and post-monsoon seasons.

	Pre-monsoon season				Post-monsoon season				
Parameters	Min	Max	Mean	% of samples exceeded the limits	Min	Max	Mean	% of samples exceeded the limits	WHO-2011
pH	6.4	8.6	6.99	2	5.7	8.6	6.34	2	6.5–8.5
EC (µS/cm)	361	3605	1481	36	79	932	400	20	1500
TDS (mg/L)	231	2307	948	92	124	1457	626	44	500
Na^+^ (mg/L)	4	255	35	2	9	286	93	10	200
K^+^ (mg/L)	1	359	49	30	1	200	29	26	12
Ca^2+^ (mg/L)	24	154	84	60	12	174	66	34	75
Mg^2+^ (mg/L)	6	74	40	26	6	104	36	20	50
TH as CaCO_3_ (mg/L)	180	1450	458	78	75	1350	282	20	–
HCO_3_^−^ (mg/L)	12	232	102	0	18	177	119	–	500
Cl- (mg/L)	28	685	207	24	36	1093	246	28	250
SO_4_^2−^ (mg/L)	6	486	95	6	6	486	152	2	250
NO_3_^−^ (mg/L)	0	631	129	62	1	215	33	24	45
F^−^ (mg/L)	0.35	4.84	1.37	24	0.17	5.22	1.07	16	1.5

**Table 2:** Chloro-alkaline Indices (CAI) and Gibb's ratio in the study area (meq/L).

	Pre-monsoon				Post-monsoon			
	CAI-1	CAI-2	Gibb's ratio I	Gibb's ratio II	CAI-1	CAI-2	Gibb's ratio I	Gibb's ratio II
Minimum	−1.36	−0.4	0.06	0.2	−1.36	−0.44	0.06	0.29
Maximum	0.91	1.85	0.75	0.94	0.86	1.92	0.88	0.94
Average	0.39	0.45	0.21	0.67	0.45	0.34	0.47	0.69

**Table 3:** Pearson correlation matrix (r^2^) of physico-chemical parameters and major ions (*N* = 50) of groundwater in pre-monsoon.

(a)													
	pH	EC (µS/cm)	TDS (mg/L)	Na^+^ (mg/L)	*K*^+^ (mg/L)	Ca^2+^ (mg/L)	Mg^2+^ (mg/L)	TH as CaCO_3_ (mg/L)	HCO_3_^−^ (mg/L)	Cl^−^ (mg/L)	SO_4_^2−^ (mg/L)	NO_3_^−^ (mg/L)	*F*^−^ (mg/L)
pH	1												
EC	−0.35*	1											
TDS	−0.35*	1.00⁎⁎	1										
Na^+^	0.04	0.54⁎⁎	0.54⁎⁎	1									
*K*^+^	−0.17	0.72⁎⁎	0.72⁎⁎	0.04	1								
Ca^2+^	−0.66⁎⁎	0.54⁎⁎	0.54⁎⁎	0.17	0.20	1							
Mg^2+^	−0.36⁎⁎	0.66⁎⁎	0.66⁎⁎	0.23	0.44⁎⁎	0.46⁎⁎	1						
HCO_3_^−^	0.05	−0.08	−0.08	−0.04	−0.01	−0.46⁎⁎	0.01	−0.23	1				
Cl^−^	−0.37⁎⁎	0.91⁎⁎	0.91⁎⁎	0.44⁎⁎	0.69⁎⁎	0.64⁎⁎	0.64⁎⁎	0.51⁎⁎	−0.22	1			
SO_4_^2−^	0.04	0.47⁎⁎	0.47⁎⁎	0.74⁎⁎	0.19	0.13	0.34*	0.14	−0.13	0.30*	1		
NO_3_^−^	−0.34*	0.84⁎⁎	0.84⁎⁎	0.40⁎⁎	0.63⁎⁎	0.57⁎⁎	0.70⁎⁎	0.64⁎⁎	−0.17	0.83⁎⁎	0.25	1	
*F*^−^	0.31*	−0.04	−0.04	0.43⁎⁎	−0.17	−0.41⁎⁎	−0.14	−0.24	0.19	−0.23	0.47⁎⁎	−0.15	1

(b)													

pH	1												
EC	−0.18	1											
TDS	−0.17	1.00⁎⁎	1										
Na^+^	−0.35*	0.56⁎⁎	0.56⁎⁎	1									
*K*^+^	−0.21	0.59⁎⁎	0.59⁎⁎	0.49⁎⁎	1								
Ca^2+^	−0.38⁎⁎	0.65⁎⁎	0.65⁎⁎	0.36*	0.26	1							
Mg^2+^	−0.26	0.62⁎⁎	0.62⁎⁎	0.37⁎⁎	0.22	0.5⁎⁎	1						
TH as CaCO_3_	0.02	0.41⁎⁎	0.41⁎⁎	−0.11	0.11	0.34*	0.32*	1					
HCO_3_^−^	−0.53⁎⁎	0.35*	0.35*	0.54⁎⁎	0.41⁎⁎	0.19	0.18	−0.13	1				
Cl^−^	−0.38⁎⁎	0.70⁎⁎	0.70⁎⁎	0.85⁎⁎	0.57⁎⁎	0.61⁎⁎	0.63⁎⁎	0.05	0.43⁎⁎	1			
SO_4_^2−^	−0.17	0.38⁎⁎	0.38⁎⁎	0.42⁎⁎	0.01	0.13	0.38⁎⁎	−0.08	0.26	0.21	1		
NO_3_^−^	−0.15	0.66⁎⁎	0.66⁎⁎	0.27	0.39⁎⁎	0.60⁎⁎	0.50⁎⁎	0.82⁎⁎	0.05	0.46⁎⁎	0.06	1	
*F*^−^	0.43⁎⁎	−0.06	−0.06	−0.13	−0.19	−0.25	−0.13	−0.09	−0.30*	−0.22	0.11	−0.12	1

⁎ Correlation is significant at the 0.05 level (2-tailed).

⁎⁎ Correlation is significant at the 0.01 level (2-tailed).

## 2 Experimental Design, Materials and Methods

### 2.1 Materials and methods

Total 50 samples that include 42 groundwater, and 8 surface water samples were collected in pre-cleaned 2-liter polyethylene bottles from the dug wells, hand pump and bore wells (groundwater), and tanks (surface water) for pre (May-2015) and post (December-2015) monsoon periods, respectively (Table 1) as per the standard procedures [8]. Fig. 1 shows the location of the collected samples. Standard physic-chemical parameters, which include pH, electric conductivity (EC), Total Dissolved solids (TDS), temperature, and salinity, were measured in-situ using the portable meters. All the major ions such as calcium (Ca^2+^), magnesium (Mg^2+^), sodium (Na^+^), potassium (K^+^), chloride (Cl^−^), sulfate (SO_4_^2−^), fluoride (F^−^), nitrate (NO_3_^−^) were analyzed using the Ion Chromatography (IC) at the center for Materials for Electronics Technology (C-MET) Laboratory, Hyderabad. Mixed standards were used to calibrate the instrument, and with the repetitive analysis, the precision of ±2% is noticed. Bicarbonate (HCO_3_^1−^) and carbonate (CO_3_^2−^) were determined using the acid-titration with endpoint detection. The charge balance is calculated between cations and anions [9,10] with a precision of ±5% for all the samples.

### 2.2 Calculation of water quality indices

#### 2.2.1 Chloro-Alkaline indices (CAI)

The ion exchange, water rock interaction mechanism is essential to know the variations in the chemical composition of groundwater [11]. The Chloro-alkaline indices CAI-1, 2 are suggested by Schoeller (1967) [12], which indicates the ion exchange between the groundwater and its host environment. The Chloro-alkaline indices used in the evaluation of the base exchange are calculated using the equations. Most of the values of chloro-alkaline indices are positive (average: 0.39, 045 and 0.45, 0.34; Table 3, Fig. 3), which explain ion-exchange reactions between groundwater and its host rocks [13].

Chloro-Alkaline Indices

(1) $$\text{CAI}1=\frac{Cl^{-}-\left(Na^{+}+K^{+}\right)}{Cl^{\_}}$$

Chloro-Alkaline Indices

(2) $$\text{CAI}2=\frac{Cl^{-}-\left(Na^{+}+K^{+}\right)}{{\text{SO}}_{4}^{-}+{\text{HCO}}_{3}^{-}+{\text{CO}}_{3}^{2-}+{\text{NO}}_{3}^{-}}$$

#### 2.2.2 Gibbs plot

The Gibbs diagram is a widely used graphical representation to establish the relationship of water composition and aquifer lithological characteristics (Gibbs 1970, Eq. (3), 4). Three distinct fields such as precipitation dominance, evaporation dominance, and rock–water interaction dominance areas are shown in the Gibbs diagram Most of the samples fall in the rock dominance area (Fig. 4).

Gibb's ratios (Gibbs, 1970) [14] are calculated with the formulae given below.

(3) $${\text{Gibb}}^{\prime }s\;\text{Ratio}\;I\;\left(\text{for}\;\text{anion}\right)=Cl^{-}/\left(Cl^{-}+\text{HC}{O_{3}}^{-}\right)$$

(4) $${\text{Gibb}}^{\prime }s\;\text{Ratio}\;\text{II}\;\left(\text{for}\;\text{cation}\right)=\left(Na^{+}+K^{+}\right)/\left(Na^{+}+K^{+}+Ca^{2+}\right)$$

Where all ions are expressed in meq/L.

#### 2.2.3 Pearson correlation analysis

The relation between the two variables is assessed by the mutual relationship between them [15, 16]. A direct correlation exists when an increase or decrease in the value of one parameter is associated with a resultant increase or decrease in the value of other parameters. The numerical values of the correlation coefficient (*r*) for the fourteen water quality parameters are tabulated (Table 3a, 3b). Based on the Pearson correlation coefficients, three groups i.e. best correlation (*r*>0.8), good correlation (*r* = 0.8 to 0.6) and moderate correlation (*r* = 0.6 to 0.5) were made. In the pre-monsoon period, the four best-correlated pairs, five good correlated pairs, and eight moderately correlated pairs and post-monsoon season shows the five best-correlated pairs, six good correlated pairs, and four moderately correlated and one negative correlated pairs (Table 4).

**Table 4:** Correlated pairs of different parameters.

	Best correlation	Good correlation	Moderate correlation	Negative correlation
	(*r*>0.8)	(*r* = 0.8–0.6)	(*r* = 0.6–0.5)	(*r*> −0.5)
Pre-monsoon	EC–TDS,	EC–K,	EC–Na,	Nil
	EC–NO_3_,	TDS–K,	EC–Ca,	
	EC–Cl,	Mg–SO_4_,	EC–TH,	
	TDS–Cl,	K–Cl,	TDS–Na,	
	TDS–NO_3_,	K–NO_3_,	TDS–Ca,	
	TDS–Cl,	Ca–Cl,	TDS–TH,	
	TDS–NO_3_	Mg–Cl,	Mg–TH,	
		Cl–NO_3_	TH—Cl	

Post-monsoon	Na–Cl,	TDS–Ca,	TDS–K,	pH–HCO_3_
	TH–NO_3_	TDS–Mg,	TDS–Na,	
		TDS–Cl,	TDS–Ca,	
		TDS–NO_3_,	TDS–Mg,	
		Ca–Cl,	K–Cl,	
		Mg–Cl	Ca–Mg	

## Declaration of Competing Interest

The authors declare that they have no known competing financial interests or personal relationships which have, or could be perceived to have, influenced the work reported in this article.

## Appendix Supplementary materials

Image, application 1
