# Supplementary material for: Groundwater quality with special reference to fluoride concentration in the granitic and basaltic contact zone of southern India
Source: Data Brief. 2020 Oct 28;33:106462. doi: 10.1016/j.dib.2020.106462 (PMC7649469; doi:10.1016/j.dib.2020.106462)
Supplement: Supplementary file 1 [file mmc1.docx]

**Table 1a Chemical analysis results of groundwater samples of the study area in pre-monsoon season**

| **ID** | **Village Name** | **Well type** | **pH** | **EC (µS/cm)** | **TDS (mg/L)** | **Na^+^**  **(mg/L)** | **K^+^**  **(mg/L)** | **Ca^2+^**  **(mg/L)** | **Mg^2+^**  **(mg/L)** | **TH as CaCO_3_ (mg/L)** | **HCO_3_^-^ (mg/L)** | **Cl^-^ (mg/L)** | **SO_4_^2-^ (mg/L)** | **NO_3_^-^ (mg/L)** | **F^-^ (mg/L)** |
| --- | --- | --- | --- | --- | --- | --- | --- | --- | --- | --- | --- | --- | --- | --- | --- |
| 1 | Basapur | HP1 | 6.9 | 2723 | 1743 | 82 | 98 | 149 | 48 | 630 | 92 | 462 | 148 | 438 | 1.13 |
| 2 | Pirla(Basapur) | HP2 | 6.9 | 1697 | 1086 | 41 | 5 | 123 | 29 | 380 | 79 | 285 | 137 | 1 | 1 |
| 3 | Jukkal | HP3 | 6.7 | 3258 | 2085 | 49 | 279 | 86 | 73 | 560 | 128 | 521 | 176 | 463 | 2.26 |
| 4 | Gundur | BW4 | 6.9 | 1642 | 1051 | 14 | 21 | 106 | 67 | 655 | 122 | 213 | 81 | 268 | 1.02 |
| 5 | Siddapur | HP5 | 7 | 825 | 528 | 7 | 47 | 48 | 44 | 385 | 140 | 32 | 122 | 38 | 1.25 |
| 6 | Mahbubpur | BW6 | 7.5 | 1223 | 783 | 10 | 4 | 90 | 61 | 520 | 61 | 130 | 56 | 231 | 1.02 |
| 7 | Dosapally | HP7 | 6.5 | 1328 | 850 | 14 | 8 | 101 | 50 | 575 | 116 | 146 | 38 | 171 | 1.5 |
| 8 | Bangarupally | BW8 | 6.9 | 909 | 582 | 8 | 3 | 74 | 52 | 430 | 198 | 51 | 68 | 59 | 1.55 |
| 9 | Wazarkhund | HP9 | 7.1 | 630 | 403 | 5 | 1 | 65 | 33 | 255 | 79 | 98 | 68 | 36 | 0.83 |
| 10 | Dondagaon | BW10 | 7.9 | 1369 | 876 | 156 | 3 | 66 | 28 | 220 | 16 | 169 | 432 | 0 | 4.84 |
| 11 | Dhannur | HP11 | 6.9 | 1322 | 846 | 32 | 3 | 74 | 39 | 350 | 92 | 103 | 148 | 40 | 1.74 |
| 12 | Padampalli | HP12 | 6.5 | 1991 | 1274 | 18 | 161 | 107 | 47 | 815 | 67 | 267 | 89 | 269 | 1.15 |
| 13 | Bijalwadi | BW13 | 6.4 | 1517 | 971 | 9 | 7 | 126 | 47 | 1450 | 65 | 185 | 48 | 215 | 1.03 |
| 14 | Bijalwadi | HP14 | 6.7 | 909 | 582 | 6 | 2 | 89 | 41 | 325 | 116 | 172 | 15 | 50 | 1.69 |
| 15 | Katalwadi | HP15 | 7 | 845 | 541 | 8 | 2 | 75 | 37 | 310 | 173 | 156 | 17 | 48 | 1.26 |
| 16 | Somur | BW16 | 6.9 | 1006 | 644 | 13 | 9 | 99 | 35 | 450 | 156 | 177 | 60 | 20 | 1.13 |
| 17 | Somur X-Road | HP17 | 6.9 | 972 | 622 | 20 | 3 | 64 | 30 | 295 | 122 | 98 | 48 | 36 | 1.63 |
| 18 | Hangarga | HP18 | 6.9 | 988 | 632 | 13 | 6 | 70 | 34 | 355 | 168 | 102 | 31 | 71 | 1.2 |
| 19 | Mahbubpur | HP 19 | 6.6 | 3605 | 2307 | 255 | 55 | 154 | 72 | 970 | 55 | 685 | 252 | 631 | 1.01 |
| 20 | Chandigaon | BW20 | 6.8 | 1702 | 1089 | 16 | 6 | 111 | 64 | 660 | 61 | 233 | 60 | 284 | 1.52 |
| 21 | pedda Ediga | HP21 | 7 | 2866 | 1834 | 24 | 359 | 86 | 59 | 490 | 116 | 450 | 111 | 372 | 0.63 |
| 22 | Longan | BW22 | 6.9 | 1630 | 1043 | 14 | 75 | 106 | 48 | 465 | 67 | 210 | 68 | 146 | 0.98 |
| 23 | Chinna Ediga | HP23 | 6.9 | 898 | 575 | 46 | 7 | 38 | 21 | 275 | 55 | 71 | 88 | 62 | 2.95 |
| 24 | Khandevallur | HP24 | 6.9 | 1370 | 877 | 21 | 16 | 76 | 46 | 395 | 122 | 152 | 27 | 74 | 0.79 |
| 25 | Muhammadabad Thanda | HP25 | 6.6 | 866 | 554 | 7 | 2 | 99 | 6 | 310 | 56 | 86 | 37 | 92 | 0.58 |
| 26 | sawargaon | HP2 6 | 6.9 | 872 | 558 | 5 | 2 | 75 | 39 | 340 | 102 | 136 | 18 | 65 | 0.79 |
| 27 | Lingam palli | HP27 | 6.9 | 913 | 584 | 6 | 55 | 64 | 27 | 360 | 153 | 188 | 23 | 11 | 0.68 |
| 28 | sawargaon | SW28 | 8.2 | 361 | 231 | 4 | 5 | 24 | 19 | 275 | 92 | 28 | 26 | 1 | 0.55 |
| 29 | sawargaon | HP 29 | 6.7 | 856 | 548 | 7 | 4 | 75 | 38 | 335 | 122 | 182 | 7 | 40 | 0.71 |
| 30 | Fathlapur | HP30 | 6.7 | 1427 | 913 | 23 | 4 | 102 | 27 | 435 | 67 | 175 | 70 | 95 | 1.32 |
| 31 | Kemraj Kallali | HP31 | 7.1 | 808 | 517 | 5 | 1 | 78 | 30 | 320 | 88 | 122 | 37 | 37 | 0.78 |
| 32 | Kemraj Kallali | HP32 | 6.8 | 3081 | 1972 | 30 | 354 | 94 | 54 | 525 | 98 | 466 | 167 | 360 | 0.65 |
| 33 | Jukkal X-Road | HP 33 | 7.3 | 1131 | 724 | 45 | 10 | 29 | 21 | 180 | 232 | 46 | 56 | 37 | 3.28 |
| 34 | Kaulas | BW34 | 6.7 | 1609 | 1030 | 14 | 95 | 101 | 34 | 255 | 49 | 198 | 70 | 157 | 1.31 |
| 35 | Takkarapalli | HP35 | 7 | 1016 | 650 | 17 | 5 | 57 | 40 | 300 | 122 | 158 | 28 | 48 | 0.96 |
| 36 | Tupdal | HP 36 | 6.9 | 845 | 541 | 18 | 2 | 57 | 27 | 225 | 98 | 148 | 20 | 44 | 1.28 |
| 37 | Jagannath palli | HP 37 | 6.7 | 998 | 639 | 12 | 2 | 90 | 30 | 465 | 102 | 138 | 33 | 34 | 1.18 |
| 38 | Santhanpally | BW38 | 6.7 | 2005 | 1283 | 42 | 4 | 86 | 54 | 500 | 122 | 311 | 98 | 187 | 1.12 |
| 39 | Bichukunda | HP39 | 6.9 | 1500 | 960 | 22 | 3 | 104 | 42 | 520 | 67 | 256 | 20 | 49 | 0.67 |
| 40 | Rajula | HP 40 | 6.7 | 1305 | 835 | 15 | 26 | 90 | 32 | 355 | 85 | 163 | 80 | 62 | 1.72 |
| 41 | Eklarg Buzurg | HP41 | 6.9 | 1391 | 890 | 14 | 3 | 97 | 63 | 565 | 55 | 108 | 240 | 45 | 1.03 |
| 42 | Eklarg Buzurg | BW42 | 6.8 | 2981 | 1908 | 21 | 311 | 99 | 73 | 635 | 92 | 550 | 149 | 300 | 0.75 |
| 43 | Nagal gaon | HP 43 | 7.4 | 2092 | 1339 | 91 | 9 | 26 | 26 | 275 | 232 | 91 | 72 | 19 | 2.75 |
| 44 | Kandarpally | HP 44 | 6.7 | 2277 | 1457 | 44 | 104 | 116 | 40 | 410 | 79 | 369 | 68 | 77 | 0.35 |
| 45 | Muhammad | HP 45 | 6.9 | 1783 | 1141 | 18 | 4 | 103 | 74 | 680 | 55 | 377 | 78 | 161 | 1.14 |
| 46 | Ladegaon | HP 46 | 7 | 1953 | 1250 | 157 | 3 | 60 | 56 | 440 | 159 | 140 | 486 | 68 | 2.3 |
| 47 | Bichukunda | BW47 | 8.1 | 642 | 411 | 13 | 3 | 31 | 26 | 320 | 116 | 38 | 52 | 5 | 2.3 |
| 48 | Bichukunda | SW48 | 8.6 | 614 | 393 | 14 | 11 | 25 | 9 | 200 | 66 | 76 | 6 | 1 | 1.16 |
| 49 | Daultapur | BW49 | 6.9 | 1072 | 686 | 10 | 2 | 95 | 11 | 335 | 12 | 147 | 47 | 68 | 0.79 |
| 50 | Bichukunda | BW50 | 6.7 | 1406 | 900 | 19 | 6 | 128 | 26 | 415 | 56 | 191 | 96 | 11 | 0.66 |

**Table 1b Chemical analysis results of groundwater samples of the study area in post-monsoon season**

| **ID** | **Village Name** | **Well type** | **pH** | **EC (µS/cm)** | **TDS (mg/L)** | **Na^+^**  **(mg/L)** | **K^+^**  **(mg/L)** | **Ca^2+^**  **(mg/L)** | **Mg^2+^**  **(mg/L)** | **TH as CaCO_3_ (mg/L)** | **HCO_3_^-^ (mg/L)** | **Cl^-^ (mg/L)** | **SO_4_^2-^ (mg/L)** | **NO_3_^-^ (mg/L)** | **F^-^  (mg/L)** |
| --- | --- | --- | --- | --- | --- | --- | --- | --- | --- | --- | --- | --- | --- | --- | --- |
| 1 | Basapur | HP | 5.8 | 675 | 1054 | 118 | 71 | 78 | 104 | 615 | 134 | 604 | 166 | 78 | 0.58 |
| 2 | Pirla(Basapur) | HP | 6 | 357 | 558 | 56 | 2 | 84 | 22 | 290 | 146 | 188 | 97 | 2 | 0.61 |
| 3 | Jukkal | HP | 6.1 | 754 | 1178 | 122 | 158 | 82 | 36 | 345 | 153 | 547 | 83 | 71 | 1.85 |
| 4 | Gundur | BW | 6.3 | 317 | 496 | 56 | 12 | 78 | 17 | 255 | 110 | 163 | 89 | 38 | 0.83 |
| 5 | Siddapur | HP | 6.1 | 198 | 310 | 14 | 12 | 35 | 41 | 250 | 116 | 50 | 72 | 6 | 1.2 |
| 6 | Mahbubpur | BW | 6.9 | 238 | 372 | 10 | 1 | 39 | 41 | 260 | 79 | 107 | 80 | 26 | 0.81 |
| 7 | Dosapally | HP | 6.2 | 238 | 372 | 26 | 8 | 45 | 21 | 195 | 92 | 107 | 79 | 21 | 0.79 |
| 8 | Bangarupally | BW | 6.1 | 238 | 372 | 22 | 1 | 57 | 29 | 255 | 128 | 107 | 80 | 16 | 0.98 |
| 9 | Wazarkhund | HP | 6.4 | 159 | 248 | 18 | 1 | 37 | 26 | 195 | 98 | 39 | 94 | 5 | 0.44 |
| 10 | Dondagaon | BW | 7.3 | 317 | 496 | 86 | 2 | 52 | 28 | 100 | 24 | 217 | 174 | 1 | 5.22 |
| 11 | Dhannur | HP | 6.9 | 541 | 846 | 32 | 3 | 74 | 39 | 330 | 92 | 103 | 148 | 40 | 1.74 |
| 12 | Padampalli | HP | 5.8 | 516 | 806 | 286 | 137 | 72 | 44 | 240 | 171 | 501 | 115 | 48 | 0.66 |
| 13 | Bijalwadi | BW | 6.4 | 621 | 971 | 9 | 7 | 126 | 47 | 1350 | 65 | 185 | 48 | 215 | 1.03 |
| 14 | Bijalwadi | HP | 5.9 | 238 | 372 | 13 | 1 | 76 | 12 | 230 | 140 | 99 | 60 | 14 | 0.59 |
| 15 | Katalwadi | HP | 6.2 | 238 | 372 | 15 | 3 | 68 | 20 | 245 | 146 | 75 | 89 | 14 | 0.88 |
| 16 | Somur | BW | 5.8 | 357 | 558 | 72 | 25 | 57 | 31 | 265 | 122 | 234 | 109 | 19 | 0.82 |
| 17 | Somur X-Road | HP | 6.1 | 238 | 372 | 48 | 2 | 39 | 18 | 165 | 128 | 75 | 100 | 10 | 1.39 |
| 18 | Hangarga | HP | 5.9 | 317 | 496 | 71 | 13 | 47 | 23 | 205 | 122 | 114 | 166 | 23 | 0.71 |
| 19 | Mahbubpur | HP | 5.7 | 794 | 1240 | 356 | 7 | 174 | 86 | 75 | 153 | 1093 | 204 | 72 | 0.6 |
| 20 | Chandigaon | BW | 5.9 | 436 | 682 | 128 | 2 | 76 | 55 | 405 | 128 | 295 | 163 | 64 | 0.64 |
| 21 | pedda Ediga | HP | 6.1 | 675 | 1054 | 252 | 200 | 70 | 24 | 265 | 177 | 469 | 181 | 58 | 0.33 |
| 22 | Longan | BW | 6 | 357 | 558 | 96 | 56 | 49 | 34 | 255 | 140 | 213 | 159 | 24 | 0.53 |
| 23 | Chinna Ediga | HP | 6.5 | 317 | 496 | 59 | 3 | 45 | 28 | 135 | 140 | 85 | 169 | 12 | 3.35 |
| 24 | Khandevallur | HP | 6.1 | 317 | 496 | 99 | 12 | 53 | 30 | 250 | 140 | 192 | 163 | 13 | 0.41 |
| 25 | Muhammadabad | HP | 5.9 | 278 | 434 | 113 | 2 | 109 | 23 | 175 | 116 | 202 | 169 | 21 | 0.17 |
| 26 | sawargaon | HP | 6.3 | 238 | 372 | 128 | 2 | 90 | 6 | 200 | 153 | 249 | 144 | 6 | 0.37 |
| 27 | Lingam palli | HP | 6.2 | 198 | 310 | 22 | 27 | 43 | 29 | 220 | 110 | 36 | 155 | 4 | 0.34 |
| 28 | sawargaon | SW | 7.4 | 79 | 124 | 18 | 2 | 16 | 32 | 170 | 55 | 46 | 145 | 1 | 0.24 |
| 29 | sawargaon | HP | 6.1 | 198 | 310 | 64 | 1 | 43 | 24 | 200 | 110 | 103 | 159 | 13 | 0.44 |
| 30 | Fathlapur | HP | 5.8 | 357 | 558 | 98 | 2 | 78 | 42 | 110 | 122 | 270 | 179 | 20 | 1.02 |
| 31 | Kemraj Kallali | HP | 6.3 | 198 | 310 | 36 | 1 | 55 | 28 | 210 | 98 | 103 | 164 | 7 | 0.45 |
| 32 | Kemraj Kallali | HP | 6 | 714 | 1116 | 188 | 172 | 82 | 32 | 325 | 153 | 479 | 204 | 73 | 0.36 |
| 33 | Jukkal X-Road | HP | 6.5 | 278 | 434 | 108 | 2 | 12 | 17 | 100 | 122 | 78 | 172 | 9 | 3.12 |
| 34 | Kaulas | BW | 5.8 | 556 | 868 | 32 | 63 | 127 | 66 | 150 | 153 | 348 | 192 | 49 | 0.44 |
| 35 | Takkarapalli | HP | 6.2 | 278 | 434 | 27 | 2 | 18 | 40 | 210 | 146 | 114 | 155 | 12 | 0.61 |
| 36 | Tupdal | HP | 6.4 | 198 | 310 | 28 | 1 | 66 | 39 | 175 | 116 | 103 | 157 | 10 | 1.09 |
| 37 | Jagannath palli | HP | 6 | 238 | 372 | 72 | 1 | 41 | 28 | 150 | 128 | 121 | 167 | 5 | 0.84 |
| 38 | Santhanpally | BW | 6.5 | 476 | 744 | 186 | 2 | 55 | 32 | 225 | 177 | 316 | 186 | 27 | 0.69 |
| 39 | Bichukunda | HP | 6.1 | 397 | 620 | 176 | 2 | 55 | 23 | 225 | 110 | 323 | 191 | 17 | 0.29 |
| 40 | Rajula | HP | 6.2 | 317 | 496 | 128 | 25 | 49 | 21 | 205 | 134 | 174 | 182 | 14 | 1.49 |
| 41 | Eklarg Buzurg | HP | 6.9 | 570 | 890 | 14 | 3 | 97 | 63 | 470 | 55 | 108 | 240 | 45 | 1.03 |
| 42 | Eklarg Buzurg | BW | 5.9 | 794 | 1240 | 312 | 135 | 90 | 78 | 285 | 177 | 987 | 203 | 69 | 0.27 |
| 43 | Nagal gaon | HP | 5.8 | 794 | 1240 | 89 | 2 | 82 | 45 | 380 | 146 | 174 | 240 | 3 | 1.08 |
| 44 | Kandarpally | HP | 6.7 | 932 | 1457 | 44 | 104 | 116 | 40 | 380 | 79 | 369 | 68 | 77 | 0.35 |
| 45 | Muhammad | HP | 6.1 | 476 | 744 | 258 | 1 | 51 | 42 | 235 | 110 | 529 | 180 | 40 | 0.75 |
| 46 | Ladegaon | HP | 7 | 800 | 1250 | 157 | 3 | 60 | 56 | 410 | 159 | 140 | 486 | 68 | 2.26 |
| 47 | Bichukunda | BW | 8.1 | 263 | 411 | 13 | 3 | 31 | 26 | 290 | 116 | 38 | 52 | 5 | 2.28 |
| 48 | Bichukunda | SW | 8.6 | 252 | 393 | 14 | 11 | 25 | 9 | 190 | 66 | 76 | 6 | 1 | 1.16 |
| 49 | Daultapur | BW | 6.5 | 159 | 248 | 28 | 1 | 53 | 20 | 210 | 18 | 96 | 162 | 19 | 1.77 |
| 50 | Bichukunda | BW | 5.8 | 317 | 496 | 56 | 4 | 66 | 48 | 165 | 110 | 217 | 182 | 20 | 0.54 |
